# Causal association between inflammatory bowel disease and 32 site-specific extracolonic cancers: a Mendelian randomization study

**DOI:** 10.1186/s12916-023-03096-y

**Published:** 2023-10-10

**Authors:** Hui Gao, Shuhao Zheng, Xin Yuan, Jiarong Xie, Lei Xu

**Affiliations:** 1 Department of Gastroenterology, The First Affiliated Hospital of Ningbo University, Ningbo, ZheJiang 315010 China; 2 Health Science Center, Ningbo University, Ningbo, 315211 Zhejiang China

**Keywords:** Mendelian randomization, Inflammatory bowel disease, Extracolonic cancer, Oral cavity cancer, Breast cancer

## Abstract

**Background:**

The risk of extracolonic cancer is increased in inflammatory bowel disease (IBD) patients, but it is not clear whether there is a causal relationship. We aimed to systematically estimate the causal relationship between IBD and extracolonic cancers.

**Methods:**

Independent genetic variants strongly associated with IBD were extracted as instrumental variables from genome-wide association study (GWAS) conducted by the International IBD Genetics Consortium including 12,882 IBD patients, 5956 Crohn’s disease (CD) patients, and 6968 ulcerative colitis (UC) patients. Three sources of cancer GWAS were selected as outcome data. Two-sample Mendelian randomization (MR) analysis was conducted to assess the causal effects of IBD on 32 extracolonic cancers. The meta-analysis was applied to assess the combined causal effect with multiple MR results.

**Results:**

IBD, CD, and UC have potential causal associations with oral cavity cancer (IBD: OR = 1.180, 95% CI: 1.059 to 1.316, *P* = 0.003; CD: OR = 1.112, 95% CI: 1.008 to 1.227, *P* = 0.034; UC: OR = 1.158, 95% CI: 1.041 to 1.288, *P* = 0.007). Meta-analysis showed a significant positive causal relationship between IBD and breast cancer (OR = 1.059; 95% CI: 1.033 to 1.086; *P* < 0.0001) as well as a potential causal relationship between CD and breast cancer (OR = 1.029; 95% CI: 1.002 to 1.055; *P* = 0.032) based on combining multiple MR results.

**Conclusions:**

This comprehensive MR analysis suggested that genetically predicted IBD, as well as its subtypes, may be a risk factor in the development of oral cavity and breast cancer.

**Supplementary Information:**

The online version contains supplementary material available at 10.1186/s12916-023-03096-y.

## Background

Inflammatory bowel disease (IBD) is a type of abnormal immune-mediated chronic intestinal inflammation with a tendency to recur throughout the lifespan, and the main subtypes include Crohn’s disease (CD) and ulcerative colitis (UC) [1]. Long-term IBD patients have been demonstrated to have an approximately 3- to 5- fold increased risk of colitis-associated colorectal cancer (CAC) [2]. According to previous reports, the pooled incidence of CAC was 5.3 cases per 1000 years of patient follow-up, accounting for 10 to 15% of the causes of death of IBD patients [2–4]. Moreover, several guidelines have proposed a standardized management regimen for IBD, including medical therapies and colonoscopic surveillance, to effectively prevent IBD from progressing to CAC [5, 6].

As a systemic disease, disorders of inflammatory bowel disease are not limited to the colorectum. Chronic systemic inflammation and immune dysregulation may also lead to an increased risk of extracolonic cancers in IBD patients [7]. A nationwide study from Finland reported that the mortality from malignant tumors in UC and CD patients was 23% and 24%, respectively, second only to cardiovascular disease at 42% and 32% [8]. Some previous studies have systematically assessed the risk association between IBD and its major subtypes (CD and UC) and cancer [9]. For example, a 30-year follow-up cohort study reported an increased risk of hematologic malignancies in patients with CD, with a standardized incidence ratio of 1.9 [10].

However, to date, there is no expert consensus or guideline specifically targeting extracolonic cancer screening or surveillance in IBD patients. Blind cancer screening and surveillance is expected to cause an increased burden of IBD management [11]. In addition, it is difficult to measure the real risk of extracolonic cancer in patients with IBD and assess causality between these two kinds of diseases due to the inevitable confounding factors of conventional epidemiologic studies.

Mendelian randomization (MR) analysis, which uses genetic variation as an instrumental variable (IV), is an emerging method for assessing causality between diseases [12, 13]. Several genome-wide association studies (GWAS) have identified multiple loci that are strongly associated with IBD and specific cancers, providing the possibility of applying MR analysis [14, 15]. An MR study previously reported a potential causal relationship between IBD, UC, and an increased risk of oral cavity cancer [16]. However, to our knowledge, no MR studies have comprehensively assessed the causal relationship between IBD, including its subtypes, and site-specific extracolonic cancers at the whole-body level.

In this study, we used the MR approach to comprehensively assess the causal association between IBD and 32 site-specific extracolonic cancers. The results of this research may provide some evidence for extracolonic cancer screening and surveillance of IBD patients.

## Methods

### Study design

**Fig. 1:**
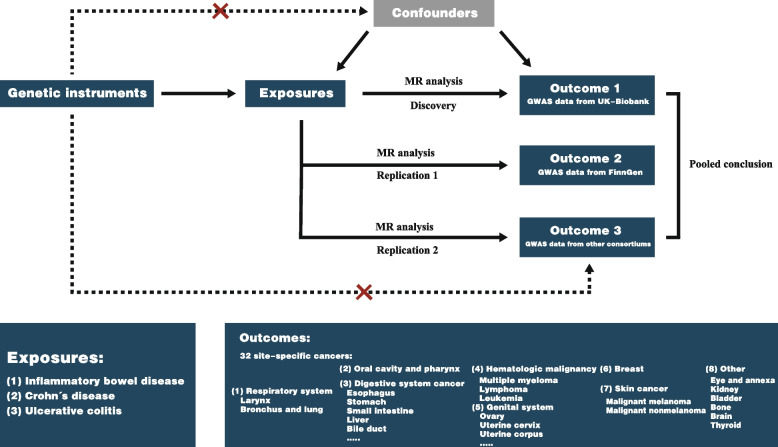
Design of this Mendelian randomization study. This study was performed with three independent cancer genome-wide association study (GWAS) datasets from the UK Biobank, FinnGen and other international consortiums. MR, Mendelian randomization

The study design is shown in Fig. 1. This MR analysis investigated the causal relationship between IBD and extracolonic cancer. Thirty-two kinds of cancer were classified into the following main categories: respiratory system cancer, oral cavity and pharynx cancer, digestive system cancer, hematologic malignancy, genital system cancer, breast cancer, skin cancer and other site cancer. We first explored the causality between IBD and 32 kinds of cancer using cancer GWAS summary data from the UK Biobank and then performed replication analyses using the FinnGen study and other GWAS conducted by international consortiums or independent teams. Finally, we combined the findings from the above MR studies. This study was conducted in accordance with the STROBE-MR guidelines (Additional file 1: Table S1).

### Data sources for IBD

A GWAS of IBD conducted by the International IBD Genetics Consortium (IIBDGC) was selected as exposure to explore the causal relationship with extracolonic cancers [17]. We selected individuals who participated in genome-wide association studies as the exposure cohort in their study, including European male and female IBD patients (12,882 cases; 21,770 controls), 5956 CD patients (14,927 controls), and 6968 UC patients (20,464 controls). Detailed information on the GWAS of the exposure is shown in Additional file 1: Table S2.

For construction of instrumental variables, genome-wide significant single-nucleotide polymorphisms (SNPs) (*P* < 5e − 08) were extracted from the GWAS summary data, and those with a longer physical distance (≥ 5000 kb) and less possibility of linkage disequilibrium (*R*^2^ < 0.01) were retained. We queried the possible phenotypes for each SNP associated with IBD, CD, and UC by PhenoScanner (http://www.phenoscanner.medschl.cam.ac.uk/) and SNPs directly associated with cancer and some commonly recognized confounding factors related to carcinogenesis were removed, such as smoking [18], alcohol consumption [19], obesity [20], and diabetes mellitus [21]. The removed SNPs and their related traits are shown in Additional file 1: Table S3. To avoid weak instrumental variable bias, we evaluated the SNP-exposure association strengths using the *F* statistic (*F* = beta/se)^2^ for each SNP. When the SNPs had an *F* value > 10, we considered a strong association between the selected IVs and exposure [22]. Finally, we averaged the *F* values of all SNPs as the overall *F* value.

### Data sources for cancer

The causal relationships between IBD and the following types of cancer were assessed in this study: malignant neoplasm of larynx, bronchus and lung; malignant neoplasm of oral cavity, pharynx; malignant neoplasm of esophagus, small intestine, stomach, pancreas, hepatocellular, hepatic bile duct and extrahepatic bile ducts (including gall bladder); multiple myeloma (MM), Hodgkin lymphoma, non-Hodgkin lymphoma, acute myelocytic leukemia, chronic myeloid leukemia and chronic lymphocytic leukemia; malignant neoplasm of ovary, uterine cervix, uterine corpus, vulva, testis and prostate; malignant neoplasm of breast; malignant melanoma of skin and malignant nonmelanoma of skin; and malignant neoplasm of eye and annexa, kidney, bladder, bone, brain, and thyroid.

Three sources of cancer GWAS studies were used to explore the causal relationship between IBD and extracolonic cancer: (i) discovery stage (Additional file 1: Table S4): We selected UK Biobank studies as the discovery cohorts because it contains the largest variety of cancer GWAS (https://pan.ukbb.broadinstitute.org/); (ii) replication stage 1 (Additional file 1: Table S5): GWAS data from the Finger r8 database including 26 type of cancers (https://r8.finngen.fi/); (iii) replication stage 2 (Additional file 1: Table S6): some GWAS studies were obtained from some large international consortiums or independent team, including the International Lung Cancer Consortium (ILCCO) (11,348 cases and 15,861 controls) [23], Pan Scan I (1896 cases and 1939 controls) [24], the Ovarian Cancer Association Consortium (OCAC, 25,509 cases and 40,941 controls) [25], a genome-wide meta-analysis on endometrial cancer (12,906 cases and 108,979 controls) [26], the Prostate Cancer Association Group to Investigate Cancer-Associated Alterations in the Genome Consortium (PRACTICAL, 79,194 cases and 61,112 controls) [27], Breast Cancer Association Consortium (BCAC, 14,910 cases and 17,588 controls, GWAS part) [28], International Agency for Research on Cancer (IARC, male: 3227 cases and 4916 controls; female: 1992 cases and 3095 controls) [29] and a GWAS of thyroid cancer conducted by Kohler A et.al (690 cases and 497 controls) [30]. Neither the UK Biobank nor FinnGen databases contain separate GWAS of oral cavity and pharynx cancers; thus, we selected a GWAS conducted by Lesseur C et al. as the discovery cohort [31]. Ethical approval and consent to participate were obtained in all original studies and were not needed for this study.

### MR analysis

We applied the inverse variance weighted (IVW) as the main estimation method to evaluate the causal relationship between exposures and outcomes. We also performed sensitivity analyses using weighted median (WM) and MR-Egger methods. The results are presented as odds ratios (ORs) and 95% confidence intervals (CIs). To assess the robustness of our findings, the heterogeneity of individual SNPs was evaluated using Cochran’s *Q* value. When there was significant heterogeneity in the MR results, we used the IVW random-effects model for correction [32]. MR pleiotropy residual sum and outlier (MR-PRESSO) method, which can detect and adjust for horizontal pleiotropy by outlier removal, was used to evaluate pleiotropy [33]. When significant pleiotropy was present, we used the MR-PRESSO method to remove outlier SNPs and calculate corrected ORs and CIs. *P* values > 0.05 were considered to indicate no significant heterogeneity or pleiotropy among the SNPs. Furthermore, we performed leave-one-out test to examine the effect of outlying and pleiotropic SNPs on causal estimates [34].

Finally, the meta-analysis was conducted to assess the combined causality between IBD and cancers from the MR results of the discovery and replication stages [35, 36]. When there was significant heterogeneity or pleiotropy, we used the corrected ORs and CIs for the meta-analysis. After removal of outlier SNPs with MR-PRESSO, MR results that still have significant heterogeneity and pleiotropy were not included into meta-analysis [37]. The choice of effect model was based on the heterogeneity of results. There was little significant heterogeneity with *I*^2^ ≤ 50%, and the fixed-effects model was applied to combine the results. For *I*^2^ > 50%, there was great heterogeneity and we used the random-effects model to combine the results. The results of the meta-analysis were chosen as the final causality [38]. However, if only one reliable MR result was available, the final causality was based on it.

To address the issue of multiple testing, we applied a Bonferroni-corrected significance threshold, which was calculated as 0.0016 (0.05/32, for the 32 types of cancer). *P* values between 0.0016 and 0.05 were considered to indicate potential causal associations between the exposures and the outcomes. All statistical analyses were performed using “TwoSampleMR,” “MRPRESSO,” and “MungeSumstats” [39] packages of the R language (version 4.2.0). Codes used for the analysis are presented Additional file 2.

## Results

### Genetic instruments and strength

In this study, 167, 154, and 111 SNPs were used as instruments for IBD, CD, and UC, respectively. The *F* statistics for each instrument exceeded 10, indicating the substantial strength of the genetic instruments used. Detailed information of SNPs used as instrumental variables for IBD, CD, and UC are shown in Additional file 1: Table S7-S12.

### Discovery results of cancer risk

**Fig. 2:**
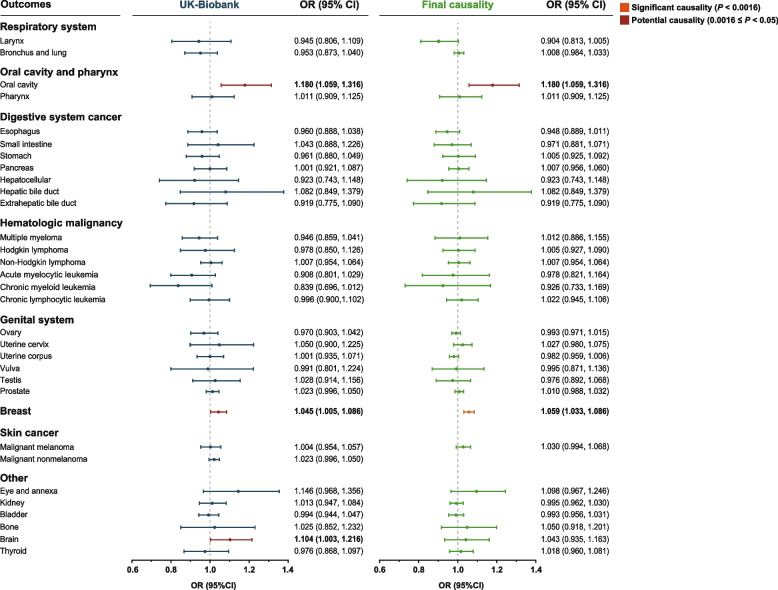
Forest plots of the association between the discovery cohort with 32 site-specific extracolonic cancers and final causality. The results of UK-Biobank database showed the corrected ORs and CIs. There is still strong heterogeneity and pleiotropy between IBD and skin malignant nonmelanoma even if we removed outlier SNPs; thus, the causality of IBD and malignant nonmelanoma could not be determined. OR, odds ratio; CI, confidence interval

First, we initially used the discovery cohort to identify causal relationship between IBD and cancers (Table 1). We found a causal relationship between IBD per unit increase in logOR and three types of extracolonic cancer that are oral cavity cancer (OR: 1.180; 95% CI: 1.059 to 1.316; *P* = 0.003), breast cancer (OR: 1.045; 95% CI: 1.005 to 1.086; *P* = 0.043), and brain cancer (OR: 1.104; 95% CI: 1.003 to 1.216; *P* = 0.043). These results suggest that IBD increases the risk of these three cancer types at the genetic level. Sensitivity analysis suggested that the association between IBD and oral cavity, brain and breast cancers was robust, showing no significant pleiotropy or heterogeneity. Leave-one-out analysis showed the similar results (Additional file 3: Figure S1). Surprisingly, we found that other than the oral cavity cancer, the occurrence of other digestive system cancers was not associated with IBD (Fig. 2).

**Table 1:** Causal effects of inflammatory bowel disease on 32 site-specific extracolonic cancer risk in UK Biobank

Phenotypes	OR_IVW_ (95% CI)	*P*_IVW_	*P*_heterogeneity_	*P*_pleiotropy_
**Respiratory system**				
Larynx	0.929 (0.789, 1.094)	0.380	0.008	0.006
Bronchus and lung	1.005 (0.956, 1.055)	0.853	0.029	0.028
**Oral cavity and pharynx**				
Oral cavity	**1.180 (1.059, 1.316)**	**0.003**	0.134	0.137
Pharynx	1.011 (0.909, 1.125)	0.836	0.724	0.722
**Digestive system cancer**				
Esophagus	0.960 (0.888, 1.038)	0.303	0.923	0.928
Small intestine	1.043 (0.888, 1.226)	0.607	0.213	0.193
Stomach	0.961 (0.880, 1.049)	0.368	0.868	0.872
Pancreas	1.001 (0.921, 1.087)	0.988	0.221	0.242
Hepatocellular	0.923 (0.743, 1.148)	0.473	0.406	0.404
Hepatic bile duct	1.082 (0.849, 1.379)	0.546	0.250	0.271
Extrahepatic bile duct	0.919 (0.775, 1.090)	0.332	0.496	0.500
**Hematologic malignancy**				
Multiple myeloma	0.938 (0.851, 1.035)	0.203	0.018	0.019
Hodgkin lymphoma	0.978 (0.850, 1.126)	0.761	0.423	0.406
Non-Hodgkin lymphoma	1.029 (0.969, 1.093)	0.343	< 0.001	0.001
Acute myelocytic leukemia	0.908 (0.801, 1.029)	0.130	0.195	0.204
Chronic myeloid leukemia	0.839 (0.696, 1.012)	0.067	0.392	0.425
Chronic lymphocytic leukemia	1.003 (0.899, 1.120)	0.952	0.002	0.003
**Genital system**				
Ovary	0.970 (0.903, 1.042)	0.411	0.714	0.700
Uterine cervix	1.050 (0.900, 1.225)	0.536	0.146	0.168
Uterine corpus	1.006 (0.938, 1.079)	0.867	0.041	0.032
Vulva	0.991 (0.801, 1.224)	0.930	0.469	0.468
Testis	1.028 (0.914, 1.156)	0.644	0.999	0.997
Prostate	1.008 (0.974, 1.044)	0.651	< 0.001	< 0.001
**Breast**	**1.045 (1.005, 1.086)**	**0.043**	0.878	0.901
**Skin cancer**				
Malignant melanoma	1.004 (0.954, 1.057)	0.871	0.074	0.051
Malignant nonmelanoma	1.030 (0.995, 1.067)	0.099	< 0.001	< 0.001
**Other**				
Eye and annexa	1.146 (0.968, 1.356)	0.115	0.483	0.478
Kidney	1.013 (0.947, 1.084)	0.701	0.482	0.478
Bladder	0.994 (0.944, 1.047)	0.821	0.106	0.098
Bone	1.025 (0.852, 1.232)	0.794	0.999	0.998
Brain	**1.104 (1.003, 1.216)**	**0.043**	0.336	0.350
Thyroid	0.976 (0.868, 1.097)	0.683	0.955	0.953

*IVW* Inverse variance weighted, *OR* Odds ratio, *CI* Confidence interval

A potentially positive association was observed for a 1-logOR increase in CD and the risk of oral cavity cancer (OR: 1.112; 95% CI: 1.008 to 1.227; *P* = 0.034) in the discovery stage. In addition, we found a potential causal relationship between CD and brain cancer (OR: 1.105; 95% CI: 1.013 to 1.205; *P* = 0.024) and malignant nonmelanoma (OR: 1.041; 95% CI: 1.009 to 1.075; *P* = 0.012). However, significant heterogeneity and pleiotropy were present (*P*_heterogeneity_ < 0.001; *P*_pleiotropy_ < 0.001) in the causal relationship between CD and malignant nonmelanoma skin cancer (Table 2).

**Table 2:** Causal effects of Crohn’s disease and ulcerative colitis on 32 site-specific extracolonic cancer risk in UK Biobank

Phenotypes	Crohn’s disease				Ulcerative colitis			
	**OR**_**IVW**_** (95% CI)**	***P***_**IVW**_	***P***_**heterogeneity**_	***P***_**pleiotropy**_	**OR**_**IVW**_** (95% CI)**	***P***_**IVW**_	***P***_**heterogeneity**_	***P***_**pleiotropy**_
**Respiratory system**								
Larynx	0.928 (0.799, 1.077)	0.090	0.046	0.051	1.025 (0.861, 1.220)	0.779	0.064	0.062
Bronchus and lung	0.997 (0.954, 1.042)	0.900	0.012	0.013	1.036 (0.985, 1.090)	0.167	0.270	0.281
**Oral cavity and pharynx**								
Oral cavity	**1.112 (1.008, 1.227)**	**0.034**	0.111	0.135	**1.158 (1.041, 1.288)**	**0.007**	0.487	0.455
Pharynx	1.019 (0.927, 1.121)	0.696	0.847	0.840	1.035 (0.924, 1.159)	0.553	0.371	0.367
**Digestive system cancer**								
Esophagus	0.982 (0.917, 1.050)	0.589	0.506	0.502	0.986 (0.906, 1.074)	0.752	0.973	0.973
Small intestine	1.037 (0.892, 1.205)	0.640	0.018	0.015	1.124 (0.949, 1.332)	0.175	0.486	0.490
Stomach	0.997 (0.924, 1.076)	0.939	0.507	0.488	0.951 (0.864, 1.046)	0.303	0.590	0.591
Pancreas	0.977 (0.910, 1.047)	0.506	0.360	0.359	1.020 (0.932, 1.116)	0.671	0.272	0.257
Hepatocellular	0.949 (0.785, 1.147)	0.586	0.387	0.366	0.908 (0.714, 1.155)	0.432	0.289	0.302
Hepatic bile duct	1.170 (0.948, 1.444)	0.144	0.257	0.285	1.020 (0.792, 1.313)	0.879	0.634	0.668
Extrahepatic bile duct	0.964 (0.831, 1.118)	0.626	0.687	0.672	0.928 (0.772, 1.116)	0.428	0.792	0.774
**Hematologic malignancy**								
Multiple myeloma	0.969 (0.894, 1.051)	0.449	0.137	0.141	0.983 (0.885, 1.091)	0.749	0.094	0.102
Hodgkin lymphoma	0.990 (0.877, 1.117)	0.867	0.669	0.673	0.965 (0.820, 1.136)	0.672	0.154	0.175
Non-Hodgkin lymphoma	1.010 (0.958, 1.065)	0.709	< 0.001	< 0.001	1.058 (0.987, 1.134)	0.110	< 0.001	< 0.001
Acute myelocytic leukemia	0.930 (0.838, 1.032)	0.170	0.524	0.508	0.991 (0.860, 1.142)	0.899	0.115	0.117
Chronic myeloid leukemia	0.889 (0.757, 1.043)	0.149	0.624	0.633	0.921 (0.747, 1.134)	0.438	0.305	0.307
Chronic lymphocytic leukemia	0.981 (0.891, 1.080)	0.697	0.004	0.006	0.967 (0.872, 1.073)	0.528	0.793	0.791
**Genital system**								
Ovary	0.951 (0.892, 1.015)	0.132	0.216	0.211	0.983 (0.909, 1.063)	0.661	0.522	0.511
Uterine cervix	1.043 (0.920, 1.184)	0.510	0.545	0.546	0.978 (0.834, 1.147)	0.786	0.808	0.807
Uterine corpus	0.989 (0.928, 1.053)	0.723	0.004	0.005	0.954 (0.885, 1.028)	0.216	0.114	0.105
Vulva	1.142 (0.947, 1.377)	0.165	0.336	0.351	0.864 (0.673, 1.109)	0.251	0.118	0.124
Testis	1.040 (0.939, 1.151)	0.451	0.996	0.997	0.983 (0.864, 1.117)	0.790	0.998	0.999
Prostate	0.998 (0.973, 1.024)	0.902	0.441	0.443	1.012 (0.973, 1.052)	0.543	< 0.001	0.001
**Breast**	1.008 (0.958, 1.061)	0.691	0.980	0.961	1.013 (0.955, 1.074)	0.675	0.692	0.697
**Skin cancer**								
Malignant melanoma	1.024 (0.978, 1.072)	0.305	0.038	0.039	1.009 (0.953, 1.069)	0.749	0.087	0.084
Malignant nonmelanoma	**1.041 (1.009, 1.075)**	**0.012**	< 0.001	< 0.001	1.012 (0.985, 1.039)	0.392	< 0.001	< 0.001
**Other**								
Eye and annexa	1.120 (0.978, 1.296)	0.129	0.561	0.564	1.092 (0.908, 1.312)	0.350	0.557	0.556
Kidney	1.033 (0.975, 1.095)	0.274	0.500	0.510	1.011 (0.937, 1.092)	0.773	0.234	0.250
Bladder	1.008 (0.965, 1.053)	0.724	0.214	0.211	0.979 (0.925, 1.035)	0.453	0.151	0.160
Bone	1.062 (0.905, 1.245)	0.460	0.978	0.987	0.900 (0.736, 1.100)	0.304	0.910	0.908
Brain	**1.105 (1.013, 1.205)**	**0.024**	0.124	0.144	1.081 (0.975, 1.198)	0.137	0.490	0.474
Thyroid	1.005 (0.907, 1.112)	0.930	0.713	0.736	0.958 (0.841, 1.091)	0.514	0.403	0.413

*IVW* Inverse variance weighted, *MR-PRESSO* Mendelian Randomization Pleiotropy Residual Sum and Outlier, *OR* oOdds ratio, *CI* Confidence interval

For UC, genetically predicted UC was potentially associated with a higher risk of oral cavity cancer in the discovery dataset (*P* = 0.007), and the OR was 1.158 (95% CI: 1.041 to 1.288). Little evidence supports a causal relationship between UC and other cancers in the discovery analyses (Table 2 and Additional file 1: Table S13).

### Replication results of cancer risk

Twenty-six cancer GWASs extracted from the FinnGen database were included in the replication study 1 (Additional file 1: Table S14-S15). There was causal effect of the presence of IBD on the increased risk of malignant melanoma skin cancer (OR: 1.056; 95% CI: 1.004 to 1.111; *P* = 0.035). We found there were causality between CD and multiple myeloma (OR: 1.089; 95% CI: 1.020 to 1.164; *P* = 0.011) and malignant nonmelanoma skin cancer (OR: 1.035; 95% CI: 1.006 to 1.064; *P* = 0.017). However, there was both significant heterogeneity and pleiotropy, indicating the causality was not robust. After we removed 11 outlier SNPs by MR-PRESSO, the MR results still showed significant heterogeneity and pleiotropy. What's more, UC was observed as the risk factor of malignant melanoma skin cancer (OR: 1.076; 95% CI: 1.016 to 1.139; *P* = 0.012) (Additional file 3: Figure S2).

Breast cancer was found to have a significant causal relationship with IBD (OR: 1.070; 95% CI: 1.035 to 1.105; *P* < 0.0001) after using the GWAS data from BCAC (Additional file 1: Table S16-S17). Causality was also confirmed in sensitivity analysis using weighted median method (OR: 1.035; 95% CI: 1.004 to 1.067; *P* = 0.0091). In addition, CD and UC was both found to have a potential causal effect on a higher risk of breast cancer in replication stage 2 (CD: OR: 1.039; 95% CI: 1.008 to 1.070; *P* = 0.013; UC: OR: 1.047; 95% CI: 1.010 to 1.086; *P* = 0.013). After three SNPs were removed by MR-PRESSO, there was still a potential causal relationship between CD and breast cancer (OR: 1.032; 95% CI: 1.003 to 1.062; *P* = 0.029). Leave-one-out analysis further demonstrated the robust of results (Additional file 3: Figure S3).

### Combined results of cancer risk from the meta-analysis

Cancer types with at least 2 reliable MR findings were integrated by meta-analysis. The summarized results of the meta-analysis are shown in Additional file 1: Table S18. We found a significant causality between IBD and breast cancer (OR = 1.059; 95% CI: 1.033 to 1.086; *P* < 0.0001) after combining two MR results from different data sources (UK Biobank and BCAC). Moreover, meta-analysis demonstrated that CD had a positive potential causal effect on breast cancer (OR = 1.029; 95% CI: 1.002 to 1.055; *P* = 0.032) after integrating the results of the UK Biobank and BCAC analyses. The causality of IBD with other extracolonic cancer was not found at the meta-analysis stage.

### Final causality of cancer risk

Pooled analysis suggested a significant positive causal relationship between IBD and breast cancer (OR = 1.059; 95% CI: 1.033 to 1.086; *P* < 0.0001), as well as a potential causal relationship between CD and breast cancer (OR = 1.029; 95% CI: 1.002 to 1.055; *P* = 0.032) based on combining multiple MR results. After performing MR analysis of the GWAS data of IIBDGC and GWAS data of oral cavity and pharynx cancer, we concluded that IBD, CD, and UC all have potential causal associations with oral cavity cancer (IBD: OR = 1.180, 95% CI: 1.059 to 1.316, *P* = 0.003; CD: OR = 1.112, 95% CI: 1.008 to 1.227, *P* = 0.034; UC: OR = 1.158, 95% CI: 1.041 to 1.288, *P* = 0.007). However, the causality may be slightly weak since it cannot be verified with cancer GWAS data from other sources. Even if outlier SNPs were identified and removed by MR-PRESSO method, all MR results between IBD or its subtypes and skin malignant non-melanoma showed significant heterogeneity and pleiotropy. Thus, no reliable results are available for us to make judgments about causality, which needs to be supplemented by follow-up studies (Table 3).

**Table 3:** Final causal relationship between inflammatory bowel disease, Crohn’s disease, ulcerative colitis and 32 site-specific extracolonic cancer

Phenotypes	**Inflammatory bowel disease**			**Crohn’s disease**			**Ulcerative colitis**		
	**Data sources**	**OR (95% CI)**	***P***	**Data sources**	**OR (95% CI)**	***P***	**Data sources**	**OR (95% CI)**	***P***
**Respiratory system**									
Larynx	UKB + FinnGen	0.904 (0.813, 1.005)	0.062	UKB + FinnGen	0.915 (0.833, 1.006)	0.067	UKB + FinnGen	0.960 (0.859, 1.072)	0.469
Bronchus and lung	UKB + FinnGen + ILCCO	1.008 (0.984, 1.033)	0.504	UKB + ILCCO	1.008 (0.965, 1.053)	0.729	UKB + FinnGen + ILCCO	1.014 (0.989, 1.040)	0.273
**Oral cavity and pharynx**									
Oral cavity	**Lesseur, C**	**1.180 (1.059, 1.316)**	**0.003**	**Lesseur, C**	**1.112 (1.008, 1.227)**	**0.034**	**Lesseur, C**	**1.158 (1.041, 1.288)**	**0.007**
Pharynx	Lesseur, C	1.011 (0.909, 1.125)	0.836	Lesseur, C	1.019 (0.927, 1.121)	0.696	Lesseur, C	1.035 (0.924, 1.159)	0.553
**Digestive system cancer**									
Esophagus	UKB + FinnGen	0.948 (0.889, 1.011)	0.103	UKB + FinnGen	0.969 (0.917, 1.024)	0.263	UKB + FinnGen	0.966 (0.899, 1.037)	0.335
Small intestine	UKB + FinnGen	0.971 (0.881, 1.071)	0.556	UKB + FinnGen	1.003 (0.921, 1.092)	0.953	UKB + FinnGen	1.009 (0.829, 1.227)	0.932
Stomach	UKB + FinnGen	1.005 (0.925, 1.092)	0.912	UKB + FinnGen	1.008 (0.960, 1.059)	0.736	UKB + FinnGen	0.993 (0.932, 1.057)	0.820
Pancreas	UKB + FinnGen + PanScan	1.007 (0.956, 1.060)	0.797	UKB + FinnGen + PanScan	0.992 (0.950, 1.036)	0.714	UKB + FinnGen + PanScan	1.029 (0.971, 1.089)	0.338
Hepatocellular	UKB	0.923 (0.743, 1.148)	0.473	UKB	0.949 (0.785, 1.147)	0.586	UKB	0.908 (0.714, 1.156)	0.432
Hepatic bile duct	UKB	1.082 (0.849, 1.379)	0.546	UKB	1.170 (0.948, 1.444)	0.144	UKB	1.020 (0.792, 1.313)	0.879
Extrahepatic bile duct	UKB	0.919 (0.775, 1.090)	0.332	UKB	0.964 (0.831, 1.118)	0.626	UKB	0.928 (0.772, 1.116)	0.428
**Hematologic malignancy**									
Multiple myeloma	UKB + FinnGen	1.012 (0.886, 1.155)	0.863	UKB + FinnGen	1.030 (0.919, 1.155)	0.611	UKB + FinnGen	1.029 (0.963, 1.100)	0.398
Hodgkin lymphoma	UKB + FinnGen	1.005 (0.927, 1.090)	0.897	UKB + FinnGen	1.013 (0.943, 1.088)	0.729	UKB + FinnGen	1.004 (0.916, 1.101)	0.932
Non-Hodgkin lymphoma	UKB	1.007 (0.954, 1.064)	0.796	UKB	0.997 (0.950, 1.047)	0.914	UKB	1.027 (0.967, 1.092)	0.385
Acute myelocytic leukemia	UKB + FinnGen	0.978 (0.821, 1.164)	0.799	UKB + FinnGen	1.035 (0.819, 1.308)	0.775	UKB + FinnGen	1.014 (0.903, 1.139)	0.814
Chronic myeloid leukemia	UKB + FinnGen	0.926 (0.733, 1.169)	0.517	UKB + FinnGen	0.907 (0.795, 1.036)	0.150	UKB + FinnGen	0.902 (0.760, 1.071)	0.240
Chronic lymphocytic leukemia	UKB + FinnGen	1.022 (0.945, 1.106)	0.581	UKB + FinnGen	0.988 (0.924, 1.057)	0.734	UKB + FinnGen	1.022 (0.905, 1.153)	0.728
**Genital system**									
Ovary	UKB + FinnGen + OCAC	0.993 (0.971, 1.015)	0.535	UKB + FinnGen + OCAC	1.004 (0.986, 1.023)	0.646	UKB + FinnGen + OCAC	0.983 (0.961, 1.006)	0.148
Uterine cervix	UKB + FinnGen	1.027 (0.980, 1.075)	0.265	UKB + FinnGen	1.031 (0.991, 1.072)	0.132	UKB + FinnGen	1.017 (0.987, 1.048)	0.258
Uterine corpus	UKB + FinnGen + O’Mara et.al	0.982 (0.959, 1.006)	0.140	FinnGen + O’Mara et.al	0.994 (0.972, 1.017)	0.598	UKB + FinnGen + O’Mara et.al	0.986 (0.961, 1.012)	0.292
Vulva	UKB + FinnGen	0.995 (0.871, 1.136)	0.940	UKB + FinnGen	1.011 (0.806, 1.267)	0.926	UKB + FinnGen	0.904 (0.779, 1.050)	0.187
Testis	UKB + FinnGen	0.976 (0.892, 1.068)	0.600	UKB + FinnGen	1.014 (0.938, 1.097)	0.727	UKB + FinnGen	0.996 (0.902, 1.101)	0.944
Prostate	UKB + FinnGen	1.010 (0.988, 1.032)	0.380	UKB + FinnGen	1.003 (0.983, 1.018)	0.972	UKB + FinnGen	1.020 (0.997, 1.045)	0.090
**Breast**	**UKB + BCAC**	**1.059 (1.033, 1.086)**	** < 0.0001**	**UKB + BCAC**	**1.029 (1.002, 1.055)**	**0.032**	UKB + FinnGen + BCAC	1.016 (0.981, 1.053)	0.372
**Skin cancer**									
Malignant melanoma	UKB + FinnGen	1.030 (0.994, 1.068)	0.105	UKB + FinnGen	1.021 (0.990, 1.053)	0.183	UKB + FinnGen	1.042 (0.979, 1.110)	0.196
Malignant nonmelanoma	None	**/**	**/**	None	**/**	**/**	None	**/**	**/**
**Other**									
Eye and annexa	UKB + FinnGen	1.098 (0.967, 1.246)	0.150	UKB + FinnGen	1.073 (0.961, 1.198)	0.212	UKB + FinnGen	1.018 (0.886, 1.170)	0.804
Kidney	UKB + FinnGen + IARC	0.995 (0.962, 1.030)	0.791	UKB + FinnGen + IARC	1.022 (0.970, 1.077)	0.417	UKB + FinnGen + IARC	0.983 (0.946, 1.021)	0.366
Bladder	UKB + FinnGen	0.993 (0.956, 1.031)	0.703	UKB + FinnGen	1.004 (0.972, 1.037)	0.799	UKB + FinnGen	0.986 (0.945, 1.028)	0.502
Bone	UKB + FinnGen	1.050 (0.918, 1.201)	0.475	UKB + FinnGen	1.005 (0.899, 1.124)	0.930	UKB + FinnGen	0.958 (0.826, 1.111)	0.572
Brain	UKB + FinnGen	1.043 (0.935, 1.163)	0.448	UKB + FinnGen	1.064 (0.992, 1.127)	0.067	UKB + FinnGen	1.039 (0.969, 1.114)	0.285
Thyroid	UKB + FinnGen + Kohler A et.al	1.018 (0.960, 1.081)	0.548	UKB + FinnGen + Kohler A et.al	1.023 (0.975, 1.074)	0.358	UKB + FinnGen + Kohler A et.al	0.990 (0.927, 1.057)	0.768

*OR* Odds ratio, *CI* Confidence interval, *ILCCO* The International Lung Cancer Consortium, *BCAC* Breast Cancer Association Consortium, *OCAC* The Ovarian Cancer Association Consortium, *IARC* International Agency for Research on Cancer

## Discussion

This was the first MR study to systematically evaluate the causal associations between IBD and 32 kinds of site-specific extracolonic cancer throughout the body. We found that IBD caused a high risk of breast cancers based on the results of an analysis combining multiple sources of cancer GWAS and CD had a potential causal relationship with breast cancer. In addition, potential causal relationship was reported between IBD, CD, UC, and oral cavity cancer. This study may provide some evidence about the risk of extracolonic cancers in patients with IBD.

Extraintestinal manifestations of IBD cover multiple systems, including the skeletal, ophthalmic, and biliary systems, such as scleritis, uveitis, ankylosing spondylitis. Many studies have provided a comprehensive overview of these manifestations and have provided priority schemes for management and treatment [40, 41]. Regular and standard endoscopic surveillance can effectively prevent the progression of IBD to CAC [42, 43], but the risk of extracolonic cancers in IBD may have been underestimated thus far. Wu et al. conducted a large cohort study using UK Biobank data, reporting that IBD patients had a 17% higher overall cancer incidence than non-IBD controls, specifically, a 33% higher incidence in nonmelanoma cancer, and a 29% higher incidence in male genital cancer [9]. Thus, it is important and urgent to clarify the association between IBD and extracolonic cancers.

We found a potential positive causal association between IBD and oral cavity cancer in our MR study (OR = 1.180, 95% CI: 1.059 to 1.316, *P* = 0.003). CD and UC, as subtypes of IBD, were also found to be potential risk factors for oral cavity cancer (CD: OR = 1.112, 95% CI: 1.008 to 1.227, *P* = 0.034; UC: OR = 1.158, 95% CI: 1.041 to 1.288, *P* = 0.007). These results are consistent with Chen’s MR study showing a potential causal relationship between IBD and oral cavity cancer (OR = 1.14, 95% CI: 1.02 to 1.27, *P* = 0.02) and between UC and oral cavity cancer (OR = 1.13, 95% CI: 1.01 to 1.27, *P* = 0.03) [16]. Our study is also consistent with several observational studies reporting IBD as a risk factor for oral cavity cancer [44, 45]. Eleven of 7294 IBD patients had biopsy-proven oral cancer during 11 years of follow-up in the USA, and standardized incidence ratio for oral cancer in patients with IBD was 9.77 (95% CI: 5.14 to 16.98) [44].

Oral pathology is a commonly reported extraintestinal manifestation of inflammatory bowel disease, with reported prevalence ranging from 0.7 to 37% in adults and 7.3 to 23% in children, such as cobble stoning mucosa, mucosal tags, and pyostomatitis vegetans [46]. These pathological changes can be regarded as site-specific manifestation of systemic inflammation in the oral cavity and chronic inflammation may lead to genetic alterations that ultimately promote carcinogenesis [47]. Additionally, the alteration in diversity of the oral microbiome in IBD patients may serve as another potential mechanism for the onset of oral cancer, based on the “gum-gut” axis hypothesis [48].

Regrettably, most of the published GWAS have put oral and pharynx cancer together as cases, but we believe it is necessary to separate these two malignancies to explore their relationship with IBD. Thus, we selected the only available GWAS, conducted by Lesseur C, as the exposure for Mendelian randomization. Given that there are not sufficient available GWAS about oral cavity and pharynx cancers, meta-analysis was not suitable to combine the results to verify this causality. However, our MR results showed no significant heterogeneity or pleiotropy, so the results could suggest, to some extent, that regular oral health screening may reduce the risk of oral cavity cancer in patients with IBD.

Pooled analysis did not show a causal relationship between IBD and gastrointestinal cancer among digestive system. Traditional observational studies have reported an association between IBD, CD, and UC and the incidence of gastrointestinal cancer [49, 50]. However, most of the previous studies are observational, and the causality remains uncertain. Compared with traditional observational studies, MR analysis can provide more reliable evidence because it is less susceptible to confounding factors and reverse causation. IBD mainly impacts the colorectum and less frequently affects the stomach and small intestine, so IBD-induced carcinogenesis of these organs is relatively rare.

This MR study ruled out direct causality between IBD and bile duct malignancy (extrahepatic and hepatic bile duct). IBD has been reported to positively affect the incidence of cholangiocarcinoma [51, 52]. However, the association may not be direct and instead occur through the mediation of primary sclerosing cholangitis (PSC). A previous MR study confirmed the causality between IBD and PSC [53], and observational studies demonstrated that IBD patients with long-term PSC have an increased risk of hepatopancreatic biliary tract cancer [52, 54]. This study assessed causality only in the UK Biobank because of limited available GWAS data, and the causal relationship between IBD and cholangiocarcinoma needs to be further investigated in MR studies with larger sample sizes or other data sources.

A large breast cancer GWAS from the UK Biobank (cases: 13,257, controls: 205,913) was used to assess the causal relationship between IBD and breast cancer, and a potential causal relationship was observed (OR: 1.045; 95% CI: 1.005 to 1.086; *P* = 0.043). After MR analysis using GWAS data from the BCAC consortium, we found a significant causal relationship between IBD and breast cancer (OR: 1.070; 95% CI: 1.035 to 1.105; *P* < 0.0001). Finally, a meta-analysis combining the results of the two cohorts further confirmed this causality (OR = 1.059; 95% CI: 1.033 to 1.086; *P* < 0.0001). Thus, we consider the association between IBD and breast cancer was robust. This result is consistent with the study of Hovde et al. published in 2017, which found that the standard incidence ratio for breast cancer was increased in CD and UC patients after stratification by sex [55].

A potential mechanism for the connection between breast cancer development and inflammatory bowel disease is that the abnormal immune environment in the breast and the systemic inflammatory reaction leads to downregulation of breast cancer resistance protein (BCRP) and upregulation of G-protein-coupled estrogen receptors (GPER) on the breast cells’ membranes. A series of subsequent pathway changes ultimately lead to the occurrence of breast cancer [56, 57].

Regrettably, there is still strong heterogeneity and pleiotropy between IBD, as well as CD and UC, and malignant nonmelanoma among SNPs in cancer data from the UK Biobank and FinnGen databases even if we removed outlier SNPs. Therefore, the causality of IBD and malignant nonmelanoma could not be determined in this study. A causal relationship between IBD and malignant melanoma was not observed in this study (OR: 1.030; 95% CI: 0.994 to 1.068; *P* = 0.105), but IBD and UC were found to have a positive relationship with skin melanoma in the FinnGen database. Previous observational studies showed that the risk of melanoma was not increased in patients diagnosed with elderly-onset IBD, but the risk of melanoma and nonmelanoma skin cancer was increased in patients diagnosed with pediatric-onset IBD [58]. If new GWASs of skin cancer with large samples are published in the future, these data could be used to verify the causality.

We found inconsistent causal relationships between IBD and some malignancies in different databases. For example, brain cancer had a suggested causal relationship with IBD in UK biobank but disappeared in the FinnGen database. Likewise, a causal relationship between multiple myeloma and CD was found in FinnGen database, but this causality did not survive in UKB. The following may explain this phenomenon. Although people recruited in FinnGen and UKB are both of European ethnicity, there are differences in the composition of the gene pool [59]. Finland is a well-established genetic isolate and a unique gene pool distinguishes Finns from other Europeans, which may lead to inconsistent causal relationships between UKB and FinnGen [60]. The discrepant results need to be interpreted with more caution. Given the “brain-gut axis” theory, IBD could lead to central nervous system disorders [61, 62]. Therefore, we cannot arbitrarily rule out the relationship between IBD and brain malignancies, which needs to be further completed by subsequent studies.

Lots of effort have been made to try to prevent instrumental variables from affecting outcomes through confounding factors or other means. After the instrumental variables extracted from the IBD GWAS data, SNPs directly related to cancer were excluded by screening in PhenoScanner website (e.g., rs6651252 was directly associated with ovarian cancer, and rs3184504 was directly associated with colorectal cancer). This effectively avoid the possibility that genetic variants directly affect the outcome. It is hard to avoid all confounding factors since carcinogenesis is multifactorial, but we removed several SNPs included in this MR analysis were likely associated with some confounders related to carcinogenesis, such as smoking, alcohol consumption, obesity, and diabetes mellitus (e.g., rs13407913 was associated with obesity, and rs6062496 was associated with smoking). The above-mentioned confounding factors related to carcinogenesis are commonly recognized, and the removal of SNPs was strictly to avoid unreliable results. Besides, we used MR-Egger and WM for supplement methods to assess the causality and MR-PRESSO was performed to remove outlier SNPs to calculate the corrected odds ratios and standard errors when there is significant pleiotropy. We believed aforementioned methods could effectively reduce potential bias and ensure the reliability of our results, to large extent.

There were several strengths of our study. First, this study is the first MR study to assess the causal association between IBD and extracolonic cancer, and the advantage of the MR design in detecting causality directly could avoid confounding factors and reverse causality compared with observational studies. Second, almost all common cancers in the whole body were included in this study. This approach provided the most systematic assessment of IBD for the risk of developing extracolonic cancers to date. Third, we conducted discovery studies and replication studies in cancer GWAS from three sources, and pooled analysis was performed after excluding results with both heterogeneity and pleiotropy to ensure the reliability of the results.

Nevertheless, we acknowledge the limitations of this study. First, due to the limited number of GWAS available or the significant heterogeneity and pleiotropy of some of the results, some conclusions come from the results of only one data source, with relatively weak robustness. Moreover, the causal relationship between IBD and breast cancer is significant in this study, but the OR is relatively small, showing that the increased risk is just modest. Third, the participants in our study were of European descent; thus, our results may not be generalizable to other ethnic populations. These findings should be validated in more diverse populations.

In this study, IBD-related instrumental variables were extracted from populations included both men and women while some cancer datasets, such as breast, prostate, and ovarian cancer, were only from single-sex cohorts. The raw IBD GWAS data selected for this study did not analyze the effect of sex dimorphism on genetic characteristics [17], but Khrom et al. recently reported some differences between sexes for disease loci location in IBD [63]. We thought that differences in gender might lead to some influence in this part of the results, which is also one of the limitations of this study.

## Conclusions

This comprehensive MR analysis suggested that IBD has a potential causal effect on oral cavity cancer and a significant causal effect on breast cancer. Regarding subtypes of IBD, there is a potential positive causal association between CD, UC, and oral cavity cancer as well as between CD and breast cancer. Our results suggest that the increased risk of these two types of extracolonic cancers in patients with IBD should not be ignored.

### Supplementary Information

**Additional file 1: Table S1. **[STROBE-MR checklist of Mendelian randomization study].** Table S2. **[Characteristics of the GWAS summary data of exposures].** Table S3. **[Removed SNPs and related traits].** Table S4. **[Cancer GWAS data information in UK Biobank].** Table S5. **[Cancer GWAS data information in FinnGen]. **Table S6. **[Cancer GWAS data information in other consortiums].** Table S7. **[SNPs used as instrumental variable for inflammatory bowel disease].** Table S8. **[Related traits of SNPs for inflammatory bowel disease]. **Table S9.** [SNPs used as instrumental variable for Crohn’s disease]. **Table S10.** [Related traits of SNPs for Crohn’s disease].** Table S11. **[SNPs used as instrumental variable for ulcerative colitis]. **Table S12.** [Related traits of SNPs for ulcerative colitis]. **Table S13.** [Sensitivity analyses in UK Biobank]. **Table S14.** [Causal effects of inflammatory bowel disease on 32 site-specific extracolonic cancer risk in FinnGen using IVW method]. **Table S15.** [Sensitivity analyses in FinnGen]. **Table S16.** [Causal effects of inflammatory bowel disease on 32 site-specific extracolonic cancer risk in other consortiums using IVW method]. **Table S17.** [Sensitivity analyses in other consortiums]. **Table S18.** [Meta-analysis results from three source of MR analysis].

**Additional file 2. **Codes used for the analysis.

**Additional file 3: Figure S1. **[Leave-one-out sensitivity test in UKB].** Figure S2. **[Leave-one-out sensitivity test in FinnGen]. **Figure S3. **[Leave-one-out sensitivity test in other consortiums]. 

## Data Availability

All of the original GWAS files are available for download; the download URL is mentioned in the Additional file 1: Table S2, Table S4-S6.

## Abbreviations

CAC: Colitis-associated colorectal cancer
CD: Crohn’s disease
CI: Confidence interval
GWAS: Genome-wide association study
IBD: Inflammatory bowel disease
IV: Instrumental variable
IVW: Inverse variance weighted
MR: Mendelian randomization
MR-PRESSO: Mendelian randomization pleiotropy residual sum and outlier
OR: Odds ratio
SNP: Single nucleotide polymorphism
UC: Ulcerative colitis
