# Supplementary material for: Causal association between inflammatory bowel disease and 32 site-specific extracolonic cancers: a Mendelian randomization study
Source: BMC Med. 2023 Oct 10;21:389. doi: 10.1186/s12916-023-03096-y (PMC10566178; doi:10.1186/s12916-023-03096-y)
Supplement: Supplementary file 2 — Additional file 2. Codes used for the analysis. [file 12916_2023_3096_MOESM2_ESM.docx]

**“TwoSampleMR” R Packages**

***#IEU open GWAS data***

library(TwoSampleMR)

exposure<- extract_instruments(

outcomes="exposure ID",

clump=TRUE, r2=0.01,

kb=5000, access_token = NULL

)

head(exposure)

dim(exposure)

t2d_out <- extract_outcome_data(

snps=exposure$SNP,

outcomes="outcome ID",

proxies = FALSE,

maf_threshold = 0.01,

access_token = NULL

)

dim(t2d_out)

mydata <- harmonise_data(

exposure_dat= exposure,

outcome_dat=t2d_out,

action= 2

)

res <- mr(mydata)

res

***# local file***

library(TwoSampleMR)

exposure<- extract_instruments(

outcomes="exposure ID",

clump=TRUE, r2=0.01,

kb=5000, access_token = NULL

)

head(exposure)

dim(exposure)

library(data.table)

t2d<-fread("file name", header = TRUE)

head(t2d)

t2d_out1 <- format_data(

dat=t2d,

type = "outcome",

snps = exposure$SNP,

header = TRUE,

phenotype_col = "phenotype",

snp_col = "variant_id",

beta_col = "beta",

se_col = "standard_error",

effect_allele_col = "effect_allele",

other_allele_col = "other_allele",

pval_col = "p_value")

mydata <- harmonise_data(

exposure_dat= exposure,

outcome_dat=t2d_out1,

action= 2

)

res <- mr(mydata)

res

*# calculate the OR and 95% CI*

generate_odds_ratios(res)

# heterogeneity test

het <- mr_heterogeneity(mydata)

het

# IVW random-effects model

mr(mydata,method_list=c('mr_ivw_mre'))

# leave-one-out test

single <- mr_leaveoneout(mydata)

mr_leaveoneout_plot(single)

**“MR-PRESSO” R Packages**

library(MRPRESSO)

mr_presso(BetaOutcome="beta.outcome",BetaExposure="beta.exposure",SdOutcome="se.outcome",SdExposure="se.exposure",

OUTLIERtest=TRUE,DISTORTIONtest=TRUE,data=mydata,NbDistribution=1000,

SignifThreshold=0.05)

**“MungeSumstats” R package**

BiocManager::install("MungeSumstats",force = TRUE)

BiocManager::install("GenomicAlignments")

BiocManager::install("SNPlocs.Hsapiens.dbSNP144.GRCh37")

BiocManager::install("BSgenome.Hsapiens.1000genomes.hs37d5")

library("MungeSumstats")

library("SNPlocs.Hsapiens.dbSNP144.GRCh37")

library("BSgenome.Hsapiens.1000genomes.hs37d5")

library(data.table)

example <-fread("filename.gz", sep =",", fill = TRUE, header=TRUE)

head(example)

MungeSumstats::format_sumstats (example, ref_genome="GRCh37")

**Codes for meta-analysis**

res<-read.csv(“filename.csv”, header=TRUE)

library(meta)

orres<- generate_odds_ratios(res)

data <- orres

data_ivw<- data[data$method=='Inverse variance weighted',]

meta <- metagen(log(or),

lower=log(or_lci95),

upper=log(or_uci95),

data=data[data$method=='Inverse variance weighted',],sm="OR",

studlab = paste(id.exposure,id.outcome),

random=T,

fixed=T,

subgroup = id.exposure)

summary(meta)
