# Supplementary material for: Causal association between inflammatory bowel disease and 32 site-specific extracolonic cancers: a Mendelian randomization study
Source: BMC Med. 2023 Oct 10;21:389. doi: 10.1186/s12916-023-03096-y (PMC10566178; doi:10.1186/s12916-023-03096-y)

Additional Figure S1

Inflammatory bowel disease (UKB)

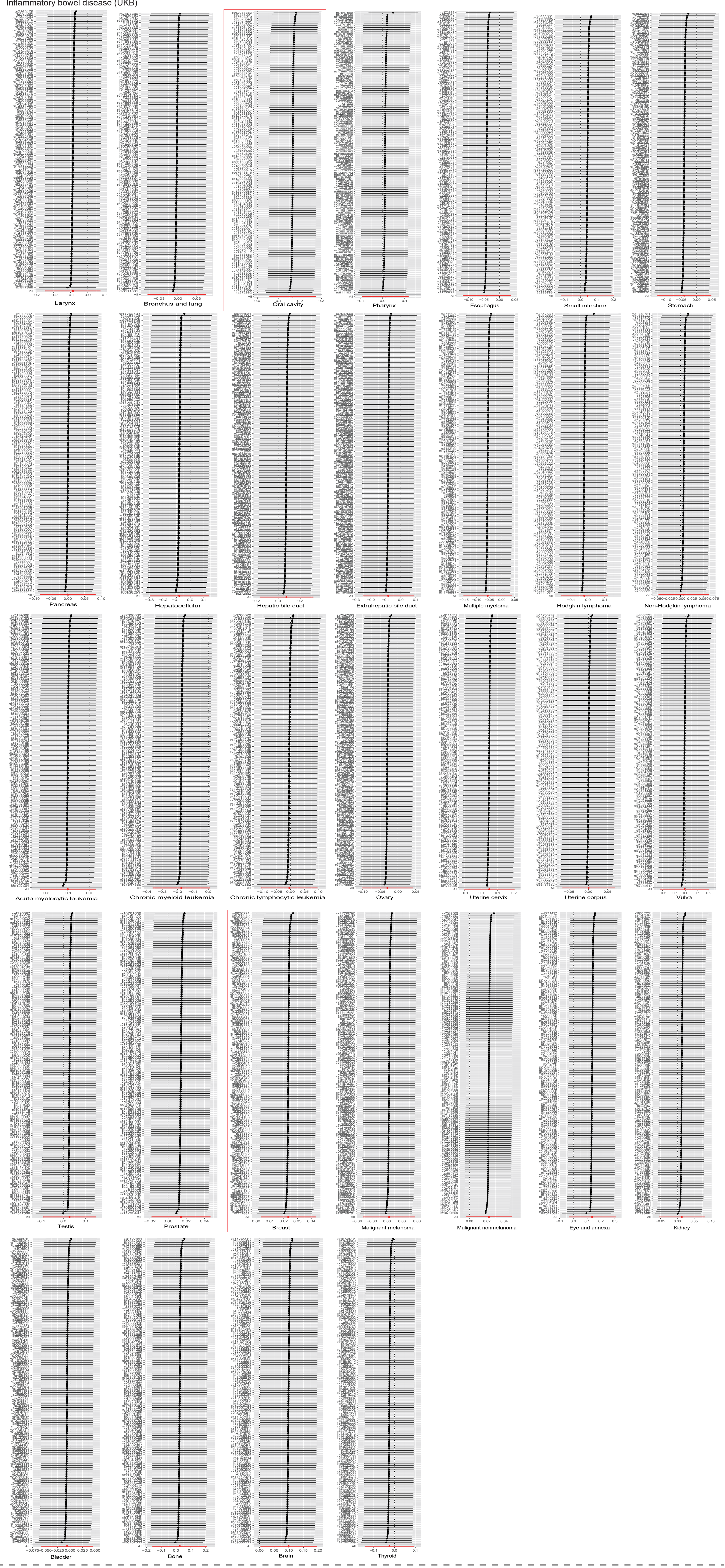

Crohn's disease (UKB)

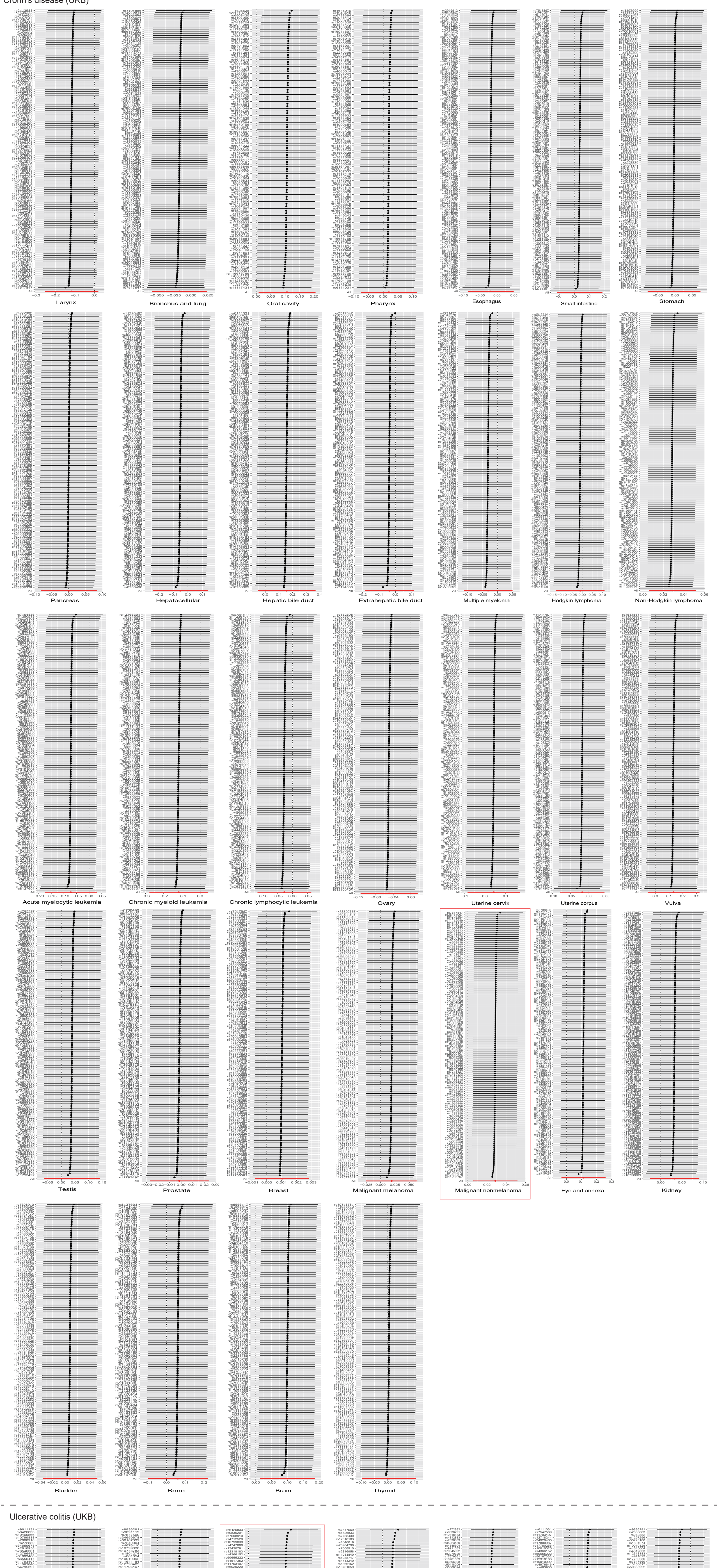

Ulcerative colitis (UKB)

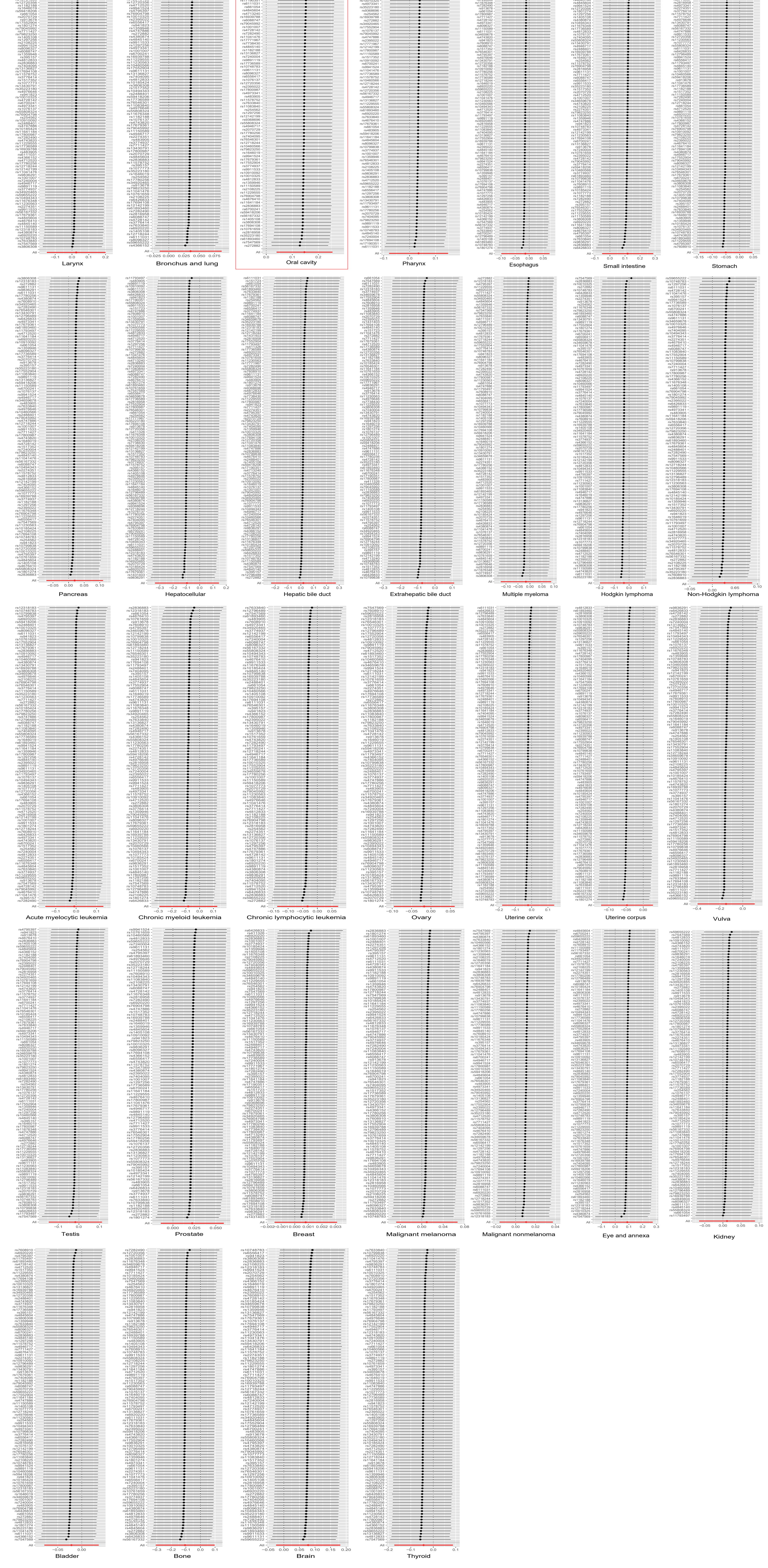

Additional Figure S2

Inflammatory bowel disease (FinnGen)

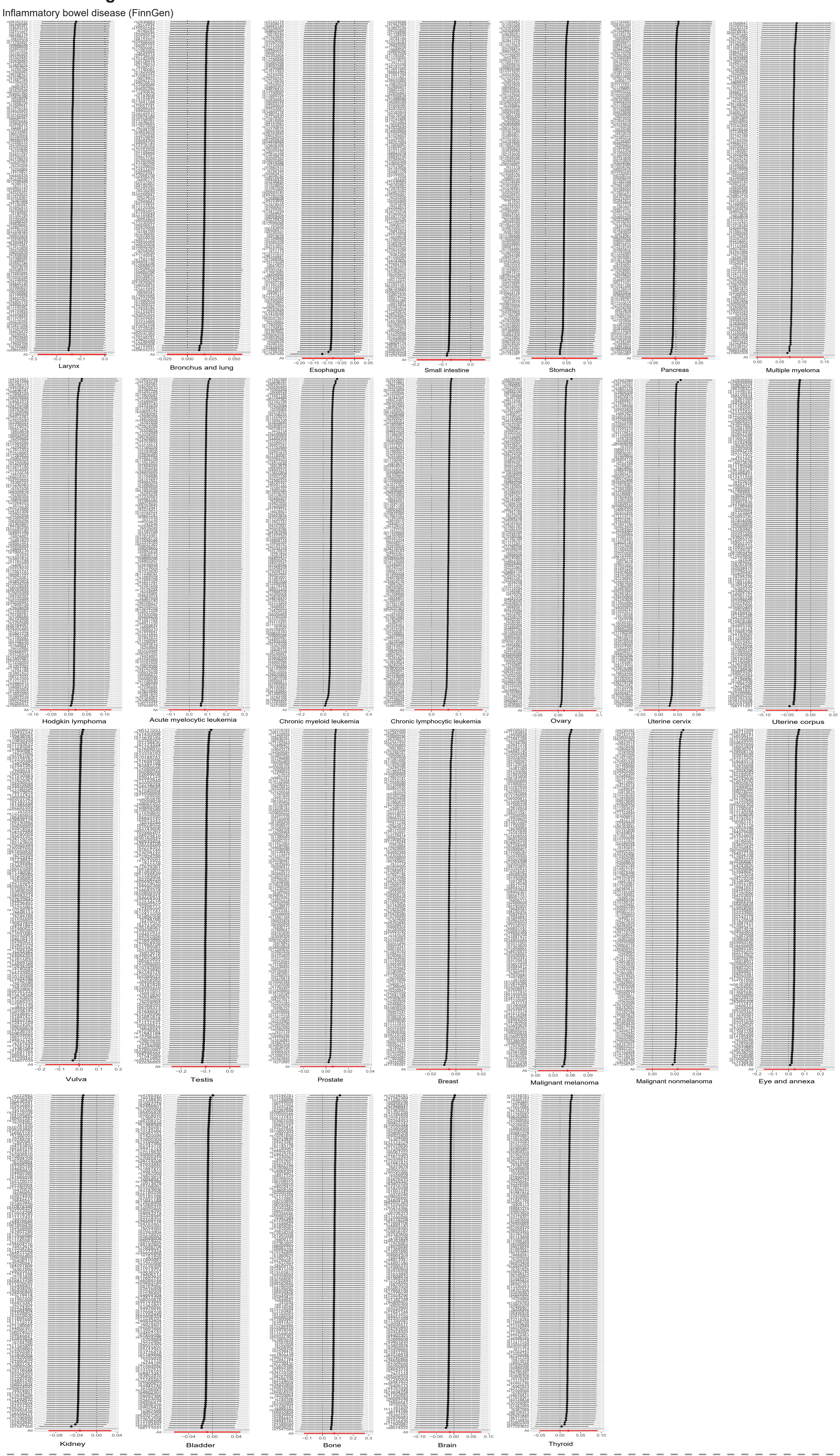

Crohn's disease (FinnGen)

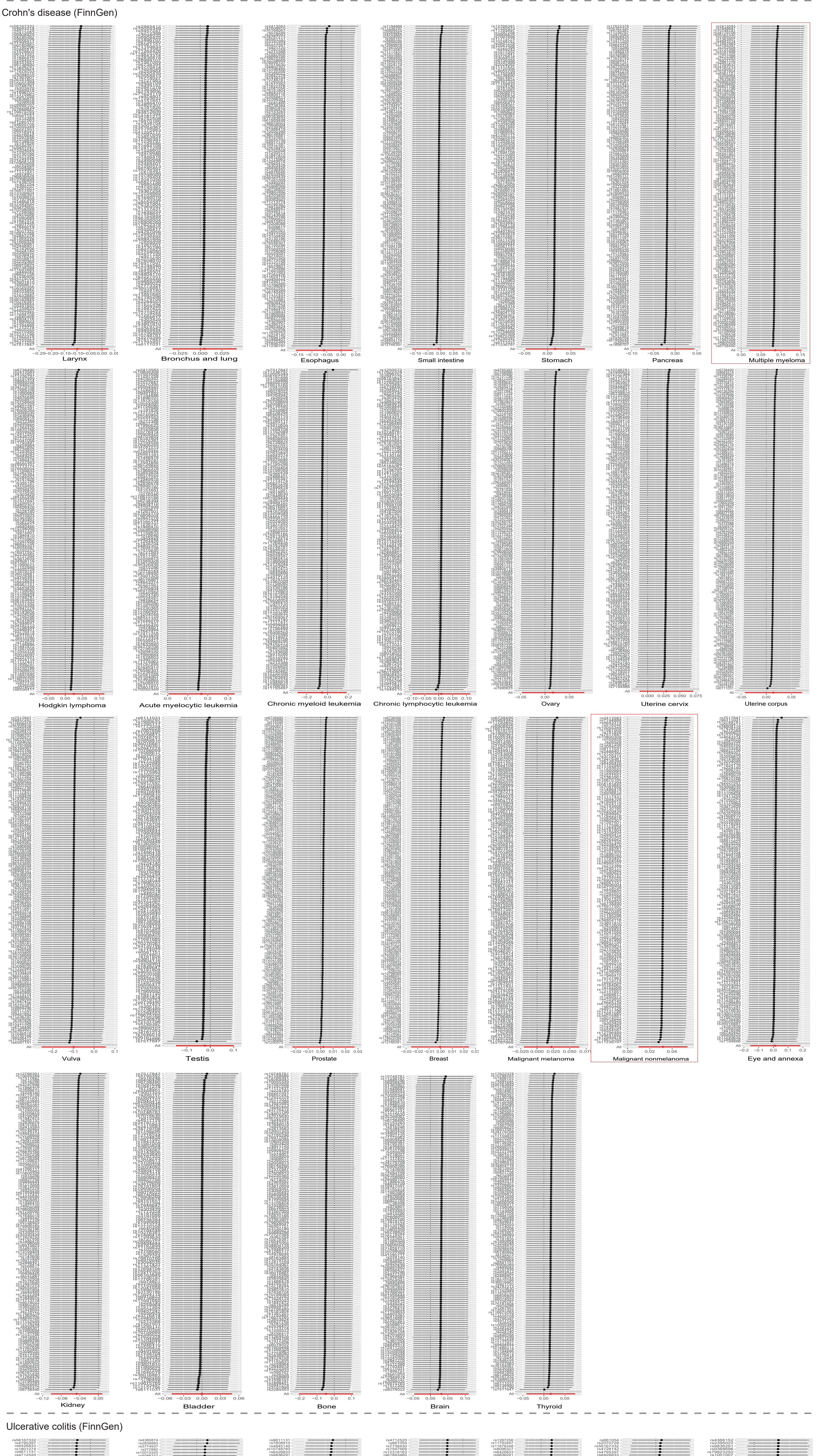

Ulcerative colitis (FinnGen)

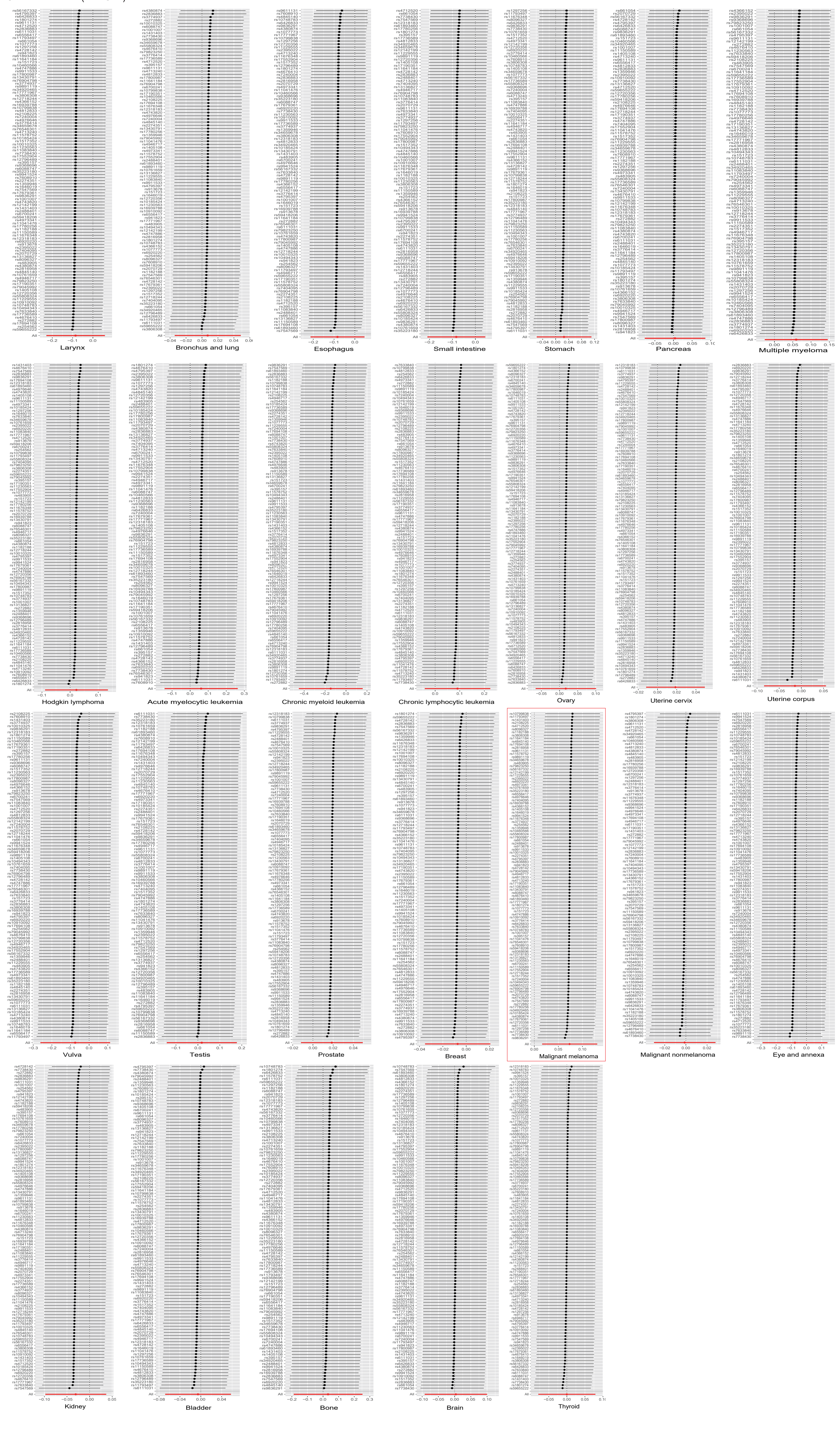

Additional Figure S3

Inflammatory bowel disease (Other consortiums)

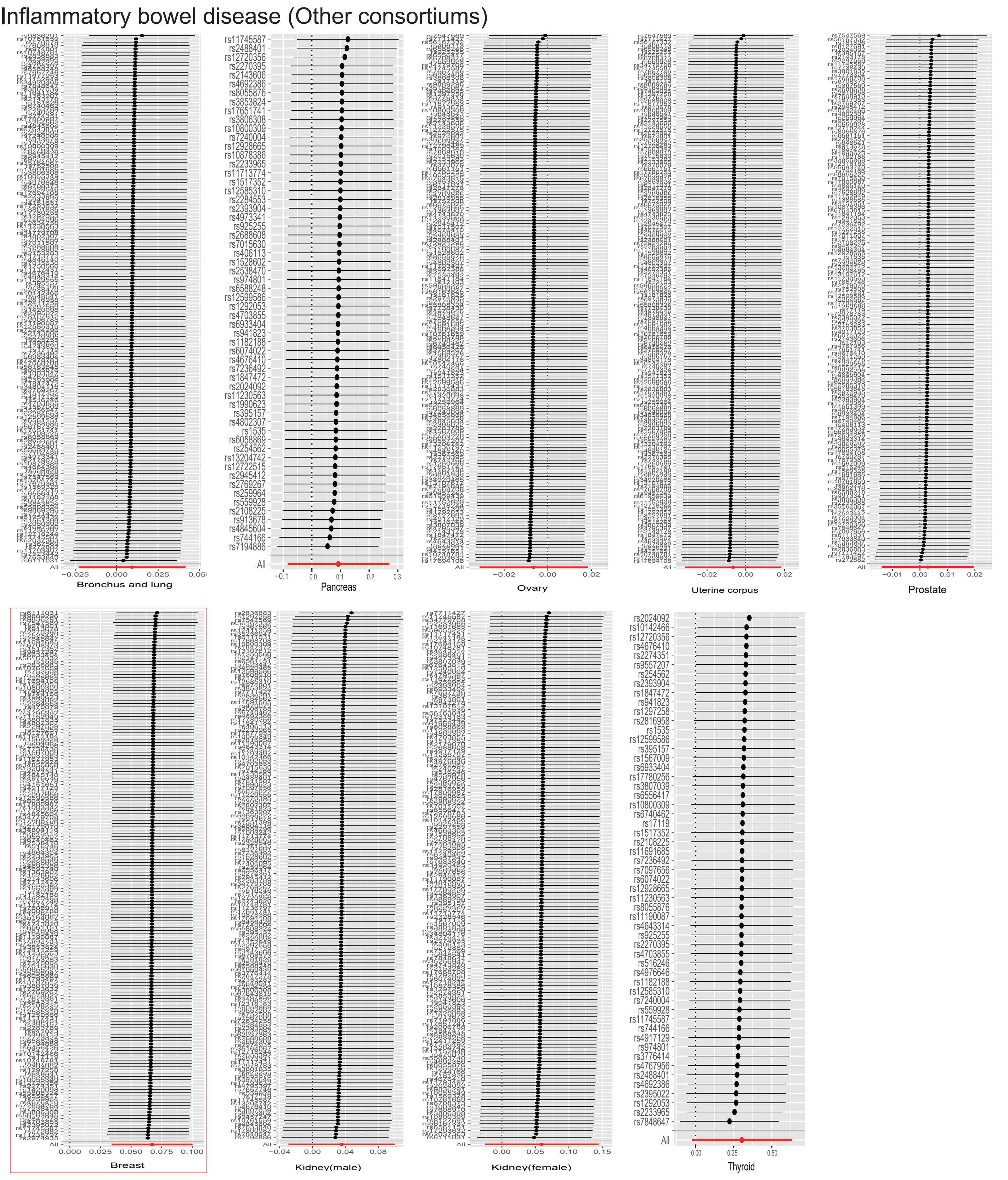

Crohn's disease (Other consortiums)

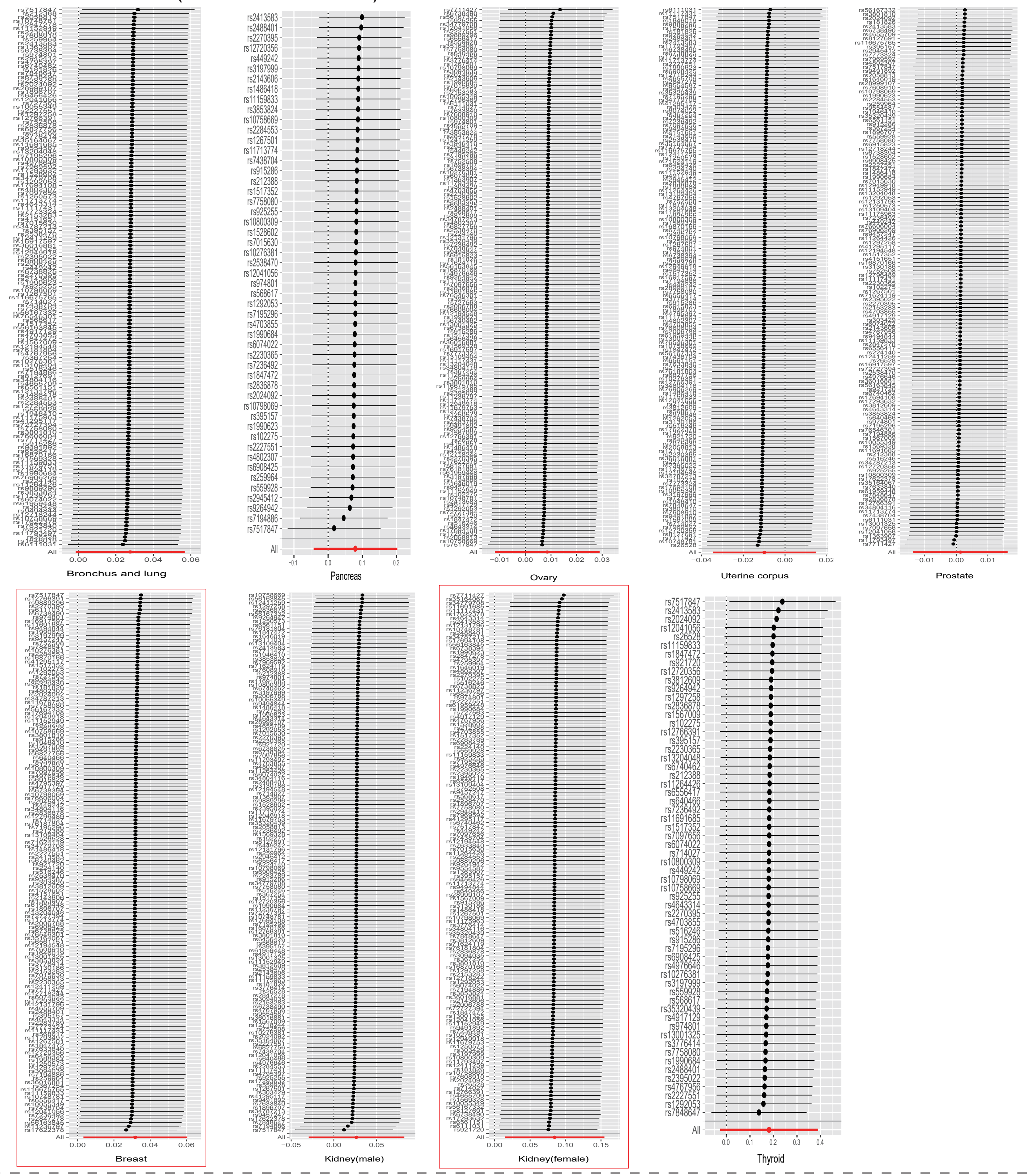

Ulcerative colitis (Other consortiums)

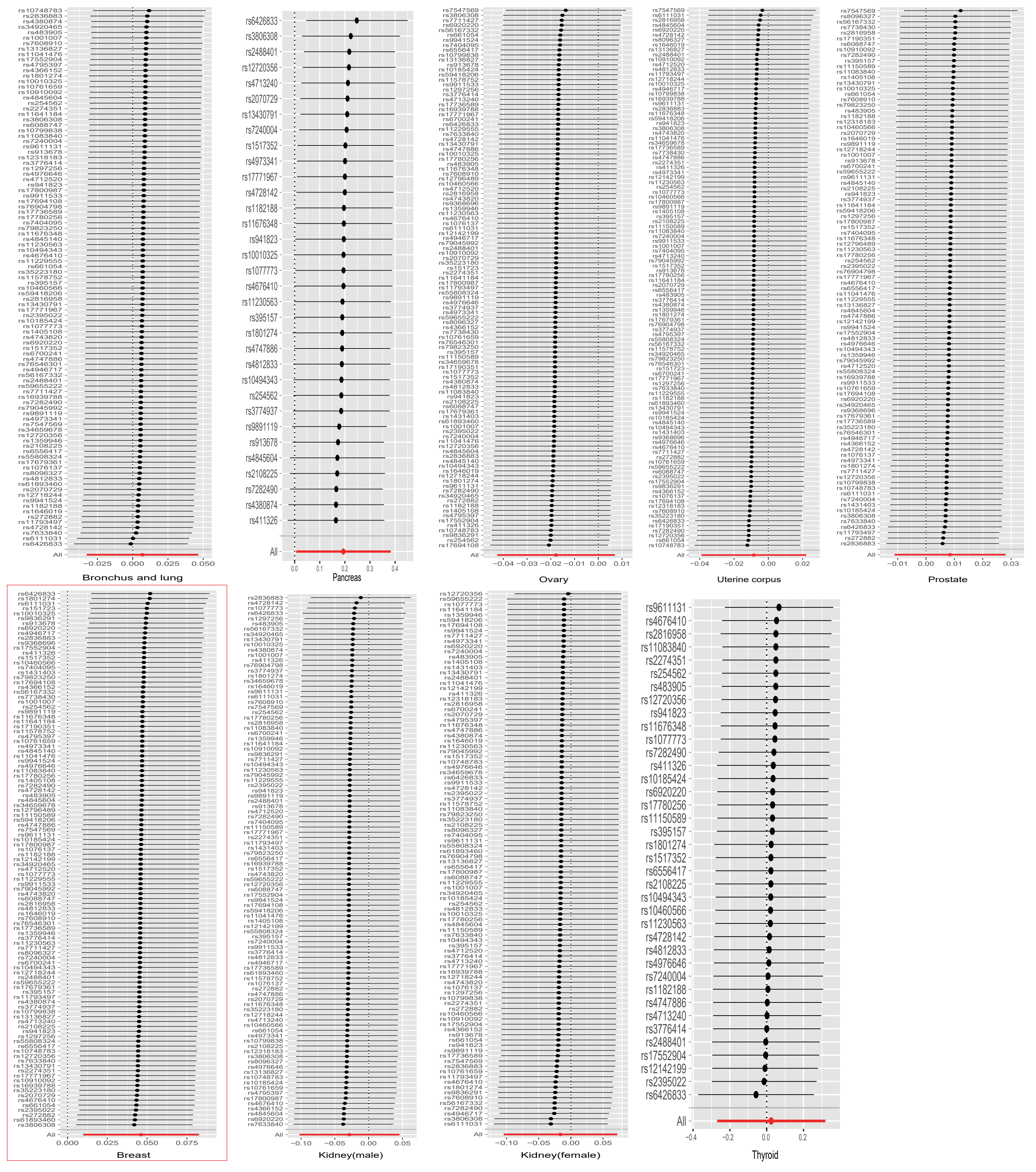

Supplement: Supplementary file 3 — Additional file 3: Figure S1. [Leave-one-out sensitivity test in UKB]. Figure S2. [Leave-one-out sensitivity test in FinnGen]. Figure S3. [Leave-one-out sensitivity test in other consortiums]. [file 12916_2023_3096_MOESM3_ESM.pdf]
